# Breakfast Cereal Consumption and Obesity Risk amongst the Mid-Age Cohort of the Australian Longitudinal Study on Women’s Health

**DOI:** 10.3390/healthcare5030049

**Published:** 2017-08-30

**Authors:** Angelica Quatela, Robin Callister, Amanda J. Patterson, Mark McEvoy, Lesley K. MacDonald-Wicks

**Affiliations:** 1 Discipline of Nutrition and Dietetics, School of Health Sciences, The University of Newcastle, Callaghan, NSW 2308, Australia; angelica.quatela@uon.edu.au (A.Q.); amanda.patterson@newcastle.edu.au (A.J.P.); 2 Priority Research Centre for Physical Activity and Nutrition, School of Biomedical Sciences and Pharmacy, The University of Newcastle, Callaghan, NSW 2308, Australia; robin.callister@newcastle.edu.au; 3 Centre for Clinical Epidemiology & Biostatistics, School of Medicine and Public Health, The University of Newcastle, Callaghan, NSW 2308, Australia; mark.mcevoy@newcastle.edu.au; 4 Hunter Medical Research Institute, New Lambton, NSW 2305, Australia

**Keywords:** obesity, muesli, porridge, breakfast cereal, ready-to-eat cereal

## Abstract

Obesity affects 27.5% of Australian women. Breakfast cereal consumption has been proposed to be protective against obesity. This study investigated the association of breakfast cereal consumption with the risk of developing obesity (Body Mass Index (BMI) ≥ 30 kg/m^2^) over 12 years among mid-age participants in the Australian Longitudinal Study on Women’s Health (ALSWH). Dietary data were obtained at S3 and obesity incidence at S4–S7. Women were excluded if: dietary data were incomplete, energy intake was <4500 or >20,000 kJ/day, or they reported being overweight or obese at S3. Logistic regressions with discrete time survival analysis investigated the association between breakfast cereal intake and incident obesity and were adjusted for: area of residency, income, smoking, physical activity, hypertension, dietary intakes and a discrete measure of time. There were 308 incident cases of obesity. Any breakfast cereal intake was not associated with incident obesity (Odds Ratio (OR): 0.92; *p* = 0.68). Oat-based cereal (OR: 0.71; *p* = 0.01), muesli (OR: 0.57; *p* = 0.00) and All-Bran (OR: 0.62; *p* = 0.01) intakes were associated with a significant reduction in obesity risk. Among this cohort, muesli on its own, or as part of oat-based cereals, and All-Bran, were associated with a reduction in obesity. This effect may be due to particular characteristics of these cereal eaters, but the relationship warrants further investigation.

## 1. Introduction

In Australia in 2011–2012, 62.8% of the adult population was found to be either overweight (BMI 25.1–29.9 kg/m^2^) or obese (BMI ≥ 30 kg/m^2^) [1] with the prevalence of obesity among women at 27.5% [2]. Worldwide, 39% of adults were overweight and 13% obese in 2014 [3]. Being overweight or obese is associated with unfavourable effects on blood cholesterol and triglycerides, insulin resistance, and blood pressure, which increase the risk of type 2 diabetes, cardiovascular diseases and ischemic stroke [3]. A higher BMI is also linked to a higher risk of some types of cancer (e.g., breast or colon cancer) [3]. A higher degree of overweight is associated with an increased risk of comorbidities related to non-communicable diseases and higher mortality [3]. Obesity has an estimated cost of about 1–3% of total health expenditure in the majority of countries and the cost reaches 5–10% in the USA [4]. 

Breakfast cereal is a grain based food product prepared from oats, corn, wheat or rice, and may undertake minimal processing, such as by drying and rolling the grain (e.g., rolled oats), or more substantial processing such as being cooked, and then flaked or puffed. Multiple grains may be mixed, and nuts and/or fruits added. Breakfast cereal is generally eaten with milk or yogurt but can be eaten in a dry state. Cereal is most frequently consumed at breakfast; however, it can also be eaten as a snack or at other meals during the day. 

Breakfast cereal consumption has been proposed to be protective against development of obesity. Two systematic reviews [5,6] found some evidence from prospective studies supporting the association of regular or frequent breakfast cereal consumption with BMI [7,8,9] or adiposity in children [10] and weight gain in men [11]. These systematic reviews examined studies in men and children, suggesting a need for more research to be conducted in women. Furthermore, Williams [6] classified the evidence base available from observational and interventional studies conducted in this area using the National Health and Medical Research Council guidelines. Williams found there is some evidence (grade B of the National Health and Medical Research Council guidelines [12,13,14]) to support an association between regular breakfast cereal consumption and a lower risk of being overweight or obese. The mechanism of action responsible for this possible protective effect of breakfast cereal on weight status is unclear [6]. One mechanism, with some weak evidence (grade C), supports an association between high-fibre breakfast cereal intake and higher satiety and lower hunger levels, which may protect against weight gain [6]. Williams, however, concluded that there is not enough evidence to differentiate the effects of different types of breakfast cereal on body weight [6]. 

It is clear that more studies are needed to investigate the effects of breakfast cereal, and different types of breakfast cereal, on the risk of developing obesity. The aim of this study was to investigate the effects of consumption of different categories of breakfast cereal on the risk of developing obesity over 12 years among participants of the mid-age cohort of the ALSWH. It is hypothesised that consumption of any breakfast cereal or consumption of high fibre breakfast cereal will be protective against the risk of developing obesity.

## 2. Materials and Methods

The ALSWH collects data every 2–3 years from four age cohorts (14,247 women aged 18–23 in 1996; 13,714 women aged 45–50 in 1996; 12,432 women aged 70–75 in 1996 and 17,015 women aged 18–23 years in 2013) in Australia with a total of 58,000 participants [15]. More information regarding the ALSWH are provided elsewhere [16].

The analyses reported in this paper were conducted in the mid-aged cohort only (women aged: 45–50 years at S1 in 1996; 47–52 years at S2 in 1998; 50–55 years at S3 in 2001; 53–58 years at S4 in 2004; 56–61 years at S5 in 2007; 59–64 years at S6 in 2010; 62–67 years at S7 in 2013). A food frequency questionnaire (FFQ) completed at S3 (2001) provided the dietary data [17,18], that were used to categorise women who consumed breakfast cereal from a list of options. Obesity incidence was the outcome of interest because it is recognised to be associated with severe risk of comorbidities [3]. Obesity data were obtained at S4 (2004) to S7 (2013), 12 years after S3 (2001).

### 2.1. Participants

This analysis was conducted in a representative sample of Australian women born between 1946–1951 who formed the mid-aged cohort of the ALSWH in 1996. These participants were randomly sampled from Medicare (the database of the Australian National Health Insurance Scheme), which includes all Australian citizens and permanent residents. The participants from remote and rural areas were sampled at twice the rate compared to participants in urban areas [16]. The ALSWH was conducted according to the guidelines laid down in the Declaration of Helsinki and all procedures involving human subjects were approved by the Human Research Ethics Committee of the University of Newcastle (approval numbers: H-076-0795 and H-2012-0256) and University of Queensland (approval numbers: 2004000224 and 2012000950) [16,19]. Written informed consent was obtained from all subjects [16,19]. Permission to access the ALSWH data for the purpose of this investigation was provided on 19 January 2015. 

### 2.2. Predictor Variables

Breakfast cereal intakes were the predictor variables and were collected at S3 in 2001 using a validated FFQ: the Dietary Questionnaire for Epidemiology Studies Version 2 (DQES-FFQv2) developed by the Cancer Council Victoria [17,18,20]. The Australian NUTTAB 95 database was used to analyse the dietary data [21]. The DQES-FFQv2 was validated by Hodge, Patterson et al. [18] in 63 women of childbearing age who completed 7-day weighed food diaries and the DQES-FFQv2; the comparison between these two dietary methods found the DQES-FFQv2 to be a valid tool to collect dietary data in adult women [18]. 

The breakfast cereal question asked: “Over the last 12 months, on average, how often did you eat the following foods?” The following breakfast cereal options were listed: (1) All-Bran; (2) Sultana Bran, Fibre Plus, Branflakes; (3) Weet Bix, Vita Brits, Weeties; (4) Cornflakes, Nutrigrain, Special K; (5) muesli; (6) porridge. The frequency of consumption options allowed for categorisation into a dichotomous variable where yes referred to any consumption higher than “never” and no referred to no consumption (“never”).

In addition, the following variables were created. A higher fibre (or whole grain) breakfast cereal category was a dichotomous variable assigned yes when at least one of these five breakfast cereal categories was consumed: porridge; muesli; All-Bran; Weet Bix, Vita Brits and Weeties; Sultana Bran, Fibre Plus and Branflakes. The oat-based cereal variable was dichotomised as “yes” when porridge or muesli or both were consumed and “no” when neither of these breakfast cereals were consumed. The wheat cereal category was “yes” if any or multiple of Weet Bix, Vita Brits or Weeties; All-Bran; Sultana Bran, Fibre Plus, or Branflakes were consumed and “no” if none of these cereals were consumed. In addition, an “any breakfast cereal” variable was derived and assigned “yes” when at least one of the six breakfast cereal categories was consumed and “no” when no breakfast cereal was consumed. 

### 2.3. Outcome Variable

The outcome variable was obesity incidence based on data from S4–S7 (2004 to 2013) up to 12 years after S3 (2001) in women who had a BMI < 25 kg/m^2^ at S3. Women who were overweight (BMI ≥ 25–29.9 kg/m^2^) or obese (BMI ≥ 30 kg/m^2^) at S3 were excluded from the analyses. BMI was calculated from self-reported height and weight and provided as a continuous variable. The obesity variable was created by categorising BMI as: <30 kg·m^−2^ (non-obese); BMI ≥ 30 kg·m^−2^ (obese). For the mid-age cohort, self-reported height and weight have been validated by Burton et al. [22] in 159 mid-aged women; this validation study found substantial agreement between the measured and self-reported height and weight and calculated BMI (Confidence Interval (CI) of the difference between the reported and the measured BMI: 0.12 (−0.13, 0.37)) [22].

### 2.4. Identification and Measurement of Confounding Factors

Area of residence, managing on income, marital status, education level, smoking, physical activity, sedentary behaviour, diabetes, impaired glucose tolerance, heart disease, hypertension, and dietary intakes (daily energy intake and fibre intake) were considered as potential confounding factors. Data for potential confounding variables were obtained from S3 with the exception of education level, which was collected at S1.

Area of residence was a dichotomous variable categorised as either urban or non-urban (remote or rural) residence. Income was evaluated as the self-reported capacity to manage on income rather than the actual monetary income level. Participant responses were assigned to one of the following two categories: income easy (it is not too bad or it is easy) or income difficult (it is impossible, it is difficult all the time, or it is difficult some of the time). Smoking status was a categorical variable composed of never-smokers, ex-smokers, or smokers. 

Physical activity status was evaluated using items from Active Australia’s 1999 National Physical Activity Survey [23]. Participants were asked to report the time spent in different categories of physical activity per week. The physical activity variable was expressed in Metabolic Equivalent Task (MET) minutes per week. A MET represents a unit of resting metabolic rate and is normally considered to be 3.5 mL oxygen/kg/min [24]. Time in activities of different intensities were multiplied by intensity-specific coefficients for each level of physical activity as follow: 3.0 * X minutes of walking, 4.0 * X minutes of moderate intensity activities, and 7.5 * X minutes of vigorous activities; the three categories were then summed to calculate total MET minutes per week [24,25]. Physical activity levels were then assigned to the following four categories: Nil/sedentary for <40 MET min/week; low for 40 to <600 MET min/week; moderate for 600 to <1200 MET min/week; high for ≥1200 MET min/week [25].

The presence or absence of hypertension, diabetes, impaired glucose tolerance or heart disease were derived from responses to this question ‘Have you ever been told by a doctor that you have’ followed by a list of medical conditions; responses were dichotomised (yes or no). The dietary factors (daily energy intake (kJ/day), and fibre (g/day) intake) were derived from the DQES-FFQv2 using the NUTTAB 95 database [21] as explained above.

### 2.5. Statistical Analyses

The participant characteristics of breakfast cereal consumers or non-consumers were compared using a two sample independent t test (for parametric distributions), a Wilcoxon Rank Sum Test (for non-parametric distributions) or a two sample t test for proportions. 

The relationship between breakfast cereal consumption (coded as yes or no consumption) at S3 and the risk of developing obesity between S4–S7 was investigated using multiple logistic regression with discrete time survival analysis models.

Confounding factors were identified as follows. A variable was a confounding factor if the p value for univariate regression analyses was ≤0.2 for the relationship between the potential confounding factor and both the predictor (breakfast cereals) and outcome (obesity incidence) variables. Variables that met these criteria were included in multivariate analyses. A variable “other breakfast cereal consumption” (coded as yes or no) was created and adjusted for in the models in order to adjust for the intake of breakfast cereals in addition to the cereal category being analysed.

Unadjusted and adjusted (for confounding factors) logistic regressions with discrete time survival analysis models were used to investigate the associations between different breakfast cereal consumption variables and subsequent incidence of obesity. Four statistical models were developed. The first (unadjusted, univariate) had only the breakfast cereal category of interest and a discrete measure of time (survey waves). The second model added non-dietary confounding factors: area of residency, income, smoking status, physical activity (METs) and hypertension. The third model adjusted for dietary confounding factors: daily energy intake, fibre, and consumption of other breakfast cereal. The fourth fully adjusted model included all confounding factors (dietary and non-dietary). Separate analyses were undertaken to investigate relationships for each of the six categories of breakfast cereals listed in the FFQ, as well as for the oat-based breakfast cereals, for wheat-based breakfast cereals, and for higher fibre breakfast cereals. The Hosmer­Lemeshow goodness-of-fit test was used to determine how well the logistic regression models fit the data. All analyses were completed using STATA version 13.

## 3. Results

A total of 11,226 women completed S3, of which 10,629 completed the DQES-FFQv2 at S3. Five thousand six hundred and thirty-two (5632, 50.2%) women were excluded because they reported being overweight or obese at S3; 854 (7.6%) women were excluded because their daily energy intake at S3 was either <4500 or >20,000 kJ/day [26,27]. Four thousand one hundred and forty-three (4143, 36.9%) women were included in the analyses.

### 3.1. Participant Characteristics

Of the 4,143 women included in the analyses, 3756 (90.7%) were breakfast cereal consumers and 387 (9.3%) were non-consumers. Of the 6486 women excluded, 5800 (89.4%) were breakfast cereal consumers and 686 (10.6%) were non-consumers. The participant characteristics of the women included in the analysis at S3 (2001) are described in Table 1 for all participants, breakfast cereal consumers, breakfast cereal non-consumers and in Table 2 for those consuming the individual types of breakfast cereals (e.g., muesli consumers). Of the total population in the analyses, approximately 60% were living in non-urban residency areas, 65% reported managing on income as either easy or not too bad, the majority of the population (62%) were never smokers, and 84% of these women engaged in some level of physical activity. Only 12% of the population reported having hypertension.

The median (inter quartile range) energy intake was 6623 (2465) kJ/day obtained from diet and 150 (501) kJ/day from alcohol. Median fibre intake was 20.0 (9.2) g/day. The energy contribution from alcohol was compared with the Acceptable Macronutrient Distribution Ranges for Australians and New Zealanders. Median alcohol intake (2.2% of energy ingested) met the recommendation of <5% [28]. Median fibre intake (20.0 g/day), however, was lower than the Recommended Dietary Intake for Australian women of 28 g/day [28].

A number of characteristics were significantly different between breakfast cereal consumers and non-consumers (Table 1). Women who did not consume breakfast cereal were significantly more likely to be smokers, experience difficulties in managing on their income, and be sedentary and less likely to engage in moderate levels of physical activity. Furthermore, those who did not eat breakfast cereal consumed less daily energy from their diet but more daily energy from alcohol and they consumed less fibre than cereal consumers. 

### 3.2. Breakfast Cereal Consumption and Risk of Obesity

A total of 308 (7.43%) incident cases of obesity were reported over 12 years of follow-up. Table 3 presents the results of the logistic regression with discrete time survival analysis models for all breakfast cereal categories both unadjusted and adjusted for confounding factors. Area of residence, income, smoking, physical activity, hypertension and dietary intakes (daily energy intake, daily fibre intake and other breakfast cereal consumption) were included in the multivariate analysis. 

The results of the fully adjusted models (adjusted for dietary and non-dietary confounding factors) are as follows. The consumption of any breakfast cereal was not associated with a significant reduction in the risk of developing obesity (OR: 0.92; CI: 0.63, 1.35; *p* = 0.68) whereas oat-based breakfast cereal was associated with a significant reduction in obesity risk (OR: 0.71; CI: 0.55, 0.90; *p* = 0.01). Wheat-based cereals (OR: 1.01; CI: 0.78, 1.31; *p* = 0.92) and higher fibre breakfast cereals (OR: 0.79; CI: 0.57, 1.10; *p* = 0.16) were not associated with a significant reduction in the risk of developing obesity. Among the six individual breakfast cereal categories, only muesli (OR: 0.57; CI: 0.43, 0.75; *p* = 0.00) and All-Bran (OR: 0.62; CI: 0.44, 0.87; *p* = 0.01) were significantly associated with a reduced risk of developing obesity. The other four breakfast cereal categories were not associated with a significant reduction in the risk of developing obesity.

## 4. Discussion

This longitudinal study investigated the effect of breakfast cereal consumption on the risk of developing obesity (BMI ≥ 30 kg/m^2^) among a large cohort of mid-aged Australian women. The consumption of any breakfast cereal, regardless of type, or higher fibre (whole grain) breakfast cereal was not protective against obesity. However, muesli on its own or as a part of the oat-based cereal group and All-Bran were significantly protective against obesity development. These findings suggest that the type of breakfast cereal consumed matters with regards to its association with obesity risk, and that few breakfast cereals may be significantly associated with a reduction in obesity risk. 

This analysis expands the evidence base in an area where data are limited. Two recent systematic reviews [5,6] summarised the evidence available from cross sectional studies, prospective studies and Randomised Controlled Trials (RCTs) regarding the association between breakfast cereal intake and weight status. With regards to prospective studies, these two reviews found some evidence supporting the association of regular or frequent breakfast cereal consumption with BMI in children (*p* = 0.020 in boys but not in girls (*p* = 0.58) over 7.5 years [9], and *p* = 0.001 in children over 3 years [7]); of BMI for age z scores and risk of being overweight (*p* < 0.01[8]); of adiposity in children (*p* = 0.008 over 3–10 years [10]) and weight gain in men (*p* = 0.007 over 13 years [11]). Also, these two reviews identified the need for more prospective studies to be conducted in this area in other population groups. This paper has contributed to the understanding of the effect of breakfast cereal intake on obesity risk in mid-aged women in Australia. Furthermore, Williams [6] found limited evidence available regarding the effect of different types of breakfast cereal on body weight. One of the strengths of our analyses was the ability to investigate the effects on obesity risk of different individual breakfast cereals and cereal categories, including categories based on fibre, oats or wheat content, thereby providing new data.

Our results found muesli to be significantly protective against obesity in mid-aged Australian women. The addition of porridge to form the oat-based cereal category weakened the protective effect and when porridge was analysed individually, it was not associated with a significant reduction in the risk of obesity. This suggests that something other than the oats in muesli, for example dried fruits, seeds or nuts, may exert a protective effect against obesity, or that there is something specific about muesli consumers that we have not been able to identify in our analysis. Another possibility is that the consumption of porridge in Australia is very seasonally based, commonly only consumed during the winter season. Therefore, the consumption of porridge may not be sufficiently consistent to provide protection against obesity. Another possibility is that porridge consumption may have not been captured to its full potential due to the season when the dietary data were collected. 

A previous study conducted in an overweight and obese population reported that the consumption of 37.5 g of oat cereal twice a day for 12 weeks, replacing usual food intake for two eating events, [29] was significantly beneficial for improving weight status and BMI compared to another cereal type (weight change over 12 weeks: control + 0.52 ± 1.74 kg, oat cereal: −2.08 ± 2.05 kg) [29]. However, this study provided evidence in an overweight and obese population only and the intake of 70 g/day may not be a realistic amount to be consumed regularly under normal circumstances. Our analyses of oat-based cereal (porridge and/or muesli) found that any intake of muesli on its own or as a part of an oat-based cereal category was significantly associated with a protective effect against obesity over 12 years in a normal weight population. 

All-Bran was also found to be significantly protective against obesity incidence in this cohort of women. The reason for this protective effect is unclear. More studies are needed to further investigate a mechanism of action and determine whether this protective effect is achieved in other populations. Although the high fibre content of All-Bran might be assumed to be the mechanism, this was controlled for in our analyses. Furthermore, higher fibre breakfast cereal was not significantly associated with a reduction in obesity risk in our cohort.

Our study has a number of strengths, the first of which is the large representative population sample analysed. Another strength is the longitudinal analysis of prospectively collected data, which lowers the risk of potential biases. Furthermore, the long period of follow-up (12 years) is an important distinction in comparison to other studies investigating breakfast cereal consumption. The robust statistical approach and the use of a validated DQES-FFQv2 are other major strengths of this study. 

There are also a number of limitations to the analyses that need to be recognised. Principally this includes the use of self-reported data. However, there is evidence from the two validation studies, one validating the DQES-FFQv2 [16,18] and the other the BMI [22], to suggest that self-reported data are adequately accurate. The analyses investigated whether there was a significant association between cereal intake collected at baseline and obesity incidence over a 12 year period. Therefore, our analyses could not account for any variations in dietary habits, such as cereal intake, over this period of time. The DQES-FFQv2 does not include information on how the breakfast cereal was prepared or when it was consumed. Similarly the categories of breakfast cereal in the DQES-FFQv2 do not capture rice-based breakfast cereal (such as “rice bubbles”) or high sugar varieties (such as ‘fruit loops’); therefore, it is not possible to evaluate whether these types of breakfast cereal would impact obesity risk.

It is important to be aware that the DQES-FFQv2 has a limited list of breakfast cereal choices, thus the bluntness of this FFQ could have impacted on our ability to evaluate breakfast cereal consumption. The list of options covers most of the common breakfast cereal types from the viewpoint of nutrition professionals; however it is possible that if the completers of this FFQ did not find their brand name breakfast cereal listed in the FFQ, they might have ticked none. The brand names of breakfast cereal options listed by the FFQ are meant to be examples of the types of breakfast cereal that would fit in these options (e.g., Sultana Bran, FibrePlus and Branflakes are examples of wheat-based high fibre cereals of which there are a number of other brand names in Australia). However, there are no explicit instructions on how to complete the breakfast cereal questions in the DQES-FFQv2, including that these examples of breakfast cereal types should be used to enable completers to categorise the breakfast cereal they consume.

## 5. Conclusions

Among mid-aged Australian women, muesli on its own, or as part of oat-based breakfast cereals, and All-Bran, but no other breakfast cereals, were associated with a reduction in obesity risk. This effect may be due to particular characteristics of these cereal eaters, but these relationships warrant further investigation

## Figures and Tables

**Table 1:** Characteristics of participants from the mid-age (2001) cohort of the Australian Longitudinal Study on Women’s Health at Survey 3 (*n* = 4143) by “any” breakfast cereal and ‘no’ breakfast cereal consumption.

	All Participants	“Any” Cereal	“No” Cereal	“Any” Cereal vs. “No” Cereal *p* Value
Sample size	*n* = 4143	90.7% (*n* = 3756)	9.3% (*n* = 387)	
Mean Age (years)	52.4 ± 1.5	52.4 ± 1.5	52.4 ± 1.4	0.9322
**Area of Residency**				
Urban	38.9%	39.0%	38.2%	0.7699
^1^ Non-urban	60.4%	60.4%	60.5%	0.9913
**Managing Income**				
^2^ Income difficult	34%	33.4%	40.1%	**0.0084**
^3^ Income easy	65.0%	65.6%	59.4%	**0.0158**
**Smoking Status**				
Never smokers	62.0%	63.2%	51.4%	**0.0000**
Ex-smokers	22.7%	22.4%	25.6%	0.1572
^4^ Current smoker	14.8%	14.1%	22.2%	**0.0000**
**Physical Activity**				
Sedentary	14.8%	14.1%	21.7%	**0.0001**
Low PA	31.2%	31.3%	20.2%	0.6555
Moderate PA	21.9%	22.3%	17.8%	**0.0412**
High PA	30.9%	31.2%	28.4%	0.2691
**Hypertension**				
no	88.2%	88.2%	88.4%	0.9351
yes	10.7%	10.7%	11.4%	0.6749
**Diet**				
* Energy intake from diet (kJ/day)	6623 ± 2465	6667 ± 2474	6052 ± 2188	**0.0000**
* Energy from alcohol (kJ/day)	150.4 ± 501.2	145.6± 475.1	202.3 ± (749.1)	**0.0234**
* Fibre (g/day)	20.0 ± 9.2	20.4 ± 9.3	16.5 ± 6.4	**0.0000**

This table summarises the data from 4143 women included in the analyses. * Data are presented as median ± interquartile range. The rest of the data are presented as mean ± Standard Deviation (SD) or % of participants. Abbreviations: PA = Physical Activity. ^1^ Non-urban: remote or rural. ^2^ Income difficult: managing income is impossible, difficult all or some of the time. ^3^ Income easy: managing income is not too bad or is easy. ^4^ Current smoker: smoking an indeterminate amount; smoking less than 10 cigarettes; smoking 10–19 cigarettes per day; smoking more than 20 cigarettes per day.

**Table 2:** Characteristics of participants from the mid-age (2001) cohort of the Australian Longitudinal Study on Women’s Health at Survey 3 (*n* = 4143) by individual breakfast cereal consumption.

	Muesli	Porridge	All-Bran	Sultana Bran, Fibre Plus and Branflakes	Weet Bix, Vita Brits and Weeties	Cornflakes, Nutrigrain and Special K
Sample size	39.0% (*n* = 1614)	50.6% *(n* = 2095)	24.6% *(n* = 1019)	30.9% (*n* = 1279)	52.2% (*n* = 2161)	41.3% (*n* =1709)
Mean Age (years)	52.4 ± 1.4	52.4 ± 1.5	52.4 ± 1.4	52.4 ± 1.5	52.4 ± 1.5	52.4 ± 1.5
**Area of Residency**						
Urban	40.5%	38.3%	40.2%	38.9%	38.1%	40.4%
^1^ Non-urban	58.8%	61.1%	59.3%	60.8%	61.1%	59.1%
**Managing Income**						
^2^ Income difficult	28.7%	34.1%	33.3%	32.8%	35.2%	35.3%
^3^ Income easy	70.4%	65.1%	65.8%	66.1%	64.0%	63.6%
**Smoking Status**						
Never smokers	66.8%	65.3%	66.3%	66.2%	63.1%	63.6%
Ex-smokers	23.2%	28.3%	21.5%	20.4%	22.4%	20.2%
^4^ Current smoker	9.6%	12.5%	11.9%	12.8%	14.2%	15.9%
**Physical Activity**						
Sedentary	8.7%	13.2%	10.7%	12.2%	13.9%	15.2%
Low PA	32.3%	31.5%	30.4%	35.1%	32.2%	32.9%
Moderate PA	23.4%	22.0%	23.0%	22.3%	23.0%	21.1%
High PA	34.5%	32.5%	34.7%	29.1%	29.9%	29.4%
**Hypertension**						
no	90.6%	88.2%	88.7%	87.9%	88.9%	88.6%
yes	8.3%	10.5%	10.0%	11.2%	10.2%	10.2%
**Diet**						
* Energy intake from diet (kJ/day)	6757 ± 2404	6812 ± 2481	6784 ± 2498	6795 ± 2597	6826 ± 2510	6891 ± 2676
* Energy from alcohol (kJ/day)	202.6 ± 508.0	129.5 ± 401.7	181.3 ± 496.4	168.9 ± 484.0	138.8 ± 432.4	132.9 ± 458.1
* Fibre (g/day)	21.6 ± 9.0	20.8 ± 9.3	23.3 ± 11.1	21.4 ± 9.7	20.6 ± 9.5	19.5 ± 9.4

This table summarises the data from 4143 women included in the analyses. * Data are presented as median ± interquartile range. The rest of the data are presented as mean ± SD or % of participants. Abbreviations: PA = Physical Activity. ^1^ Non-urban: remote or rural. ^2^ Income difficult: managing income is impossible, difficult all or some of the time. ^3^ Income easy: managing income is not too bad or is easy. ^4^ Current smoker: smoking an indeterminate amount; smoking less than 10 cigarettes; smoking 10–19 cigarettes per day; smoking more than 20 cigarettes per day.

**Table 3:** Results of the logistic regression models examining the effects of consuming breakfast cereal at Survey 3 on the risk of developing obesity from Surveys 4–7.

Breakfast Cereal Mid 3 (Yes and No)	Model 1 *		Model 2 *		Model 3 *		Model 4 *	
	Odds Ratio (CI)	*p* value	Odds Ratio (CI)	*p* value	Odds Ratio (CI)	*p* value	Odds Ratio (CI)	*p* value
Any breakfast cereal	0.76 (0.53, 1.09)	0.13	0.87 (0.59, 1.27)	0.46	0.87 (0.60, 1.25)	0.45	0.92 (0.63, 1.35)	0.68
Muesli	0.46 (0.35, 0.60)	**0.00**	0.55 (0.42, 0.73)	**0.00**	0.49 (0.31, 0.62)	**0.00**	0.57 (0.43, 0.75)	***0.00***
Porridge	0.77 (0.62, 0.97)	**0.03**	0.81 (0.64, 1.03)	0.08	0.80 (0.64, 1.01)	0.07	0.81 (0.64, 1.03)	0.09
All-Bran	0.56 (0.41, 0.76)	**0.00**	0.59 (0.43, 0.82)	**0.00**	0.65 (0.47, 0.89)	**0.01**	0.62 (0.44, 0.87)	***0.01***
Sultana Bran, Fibre Plus and Branflakes	0.86 (0.67, 1.10)	0.24	0.91( 0.70, 1.19)	0.50	0.93 (0.72,1.20)	0.57	0.94 (0 .72, 1.23)	0.68
Weet Bix, Vita Brits and Weeties	0.99 (0.79, 1.24)	0.91	1.04 (0.82, 1.32)	0.76	1.04 (0.82, 1.30)	0.77	1.07 (0.84, 1.36)	0.58
Cornflakes, Nutrigrain and Special K	1.35 (1.08 ,1.70)	**0.01**	1.27 (1.00, 1.61)	**0.05**	1.29 (1.03, 1.63)	**0.03**	1.26 (0.99, 1.60)	0.07
Oat-based breakfast cereal	0.62 (0.49, 0.78)	**0.00**	0.70 (0.55, 0.89)	**0.00**	0.66 (0.52, 0.83)	***0.00***	0.71 (0.55, 0.90)	***0.01***
Wheat-based breakfast cereal	0.89 (0.70, 1.13)	0.34	0.96 (0.75, 1.24)	0.77	0.99 (0.77, 1.27)	0.94	1.01 (0.78, 1.31)	0.92
Higher fibre (or whole grain) breakfast cereal	0.64 (0.48, 0.86)	**0.00**	0.77 (0.56, 1.06)	0.11	0.71 (0.52, 0.97)	**0.03**	0.79 (0.57, 1.10)	0.16

* Model 1 univariate model with predictor variable, outcome and a discrete measure of time (wave). Model 2 with predictor variable, outcome, discrite measure of time and adjusted for non dietary counfoundiing factors (smoking, managing income, area of residency, physical activity and hypertension). Model 3 with predictor variable, outcome, discrete measure of time and adjusted for dietary counfounding factors (daily energy intake, fibre and other breakfast cereals consumption). Model 4 with predictor variable, outcome, discrete measure of time and adjusted for dietary and non dietary counfouding factors.
